# The Face of Left-Wing Dissent: Progressives and Traditional Liberals Generate Divergently Negative and Positive Representations of J.K. Rowling

**DOI:** 10.1177/01461672251378189

**Published:** 2025-11-05

**Authors:** Elena A. Magazin, Geoffrey Haddock, Travis Proulx

**Affiliations:** 1 Cardiff University, UK

**Keywords:** political psychology, social cognition, social judgment, gender identity, tolerance

## Abstract

Recent perspectives have challenged the view that left-wingers are less likely to derogate out-group members. Building on research demonstrating heightened out-group derogation by Progressive (vs. Traditional Liberal) left-wingers, we examined whether Progressives and Traditional Liberals differentially tolerate in-group dissenters on the issue of gender identity. Using reverse correlation, Study 1 found that the Progressive-generated face of J.K. Rowling (a prominent gender critical left-winger) was evaluated negatively, while the Traditional Liberal–generated face was evaluated positively. Study 2 found that in both abstract (general description) and concrete (public figure) presentations, faces of left-wing in-group members expressing gender critical views were judged more negatively than self-identification faces, with Progressive-generated gender critical faces evaluated more negatively than Traditional Liberals–generated faces. Replicating Study 1, the Rowling face generated by Traditional Liberals was evaluated positively. These findings suggest an ideological asymmetry of derogation within left-wing kinds, offering evidence of opposing representations of a dissenting in-group target.

## Introduction

*If we can’t erase Rowling, what can we do instead?* [. . .] *we can consider accepting that the microaggressions we may have noticed in Rowling’s books themselves, were, perhaps, warning signs obscured by a benevolent, liberal exterior.* (Romano, 2020, para. 26)

Much has been said about the “culture war” consuming Western political discourse, with adherents to right- and left-wing ideological perspectives squaring off against each other. On issues ranging from workplace diversity initiatives, conflicts in the Middle East and climate change, these conflicts are often construed as the ongoing struggle of left vs. right to shape cultural norms (Swedlow et al., 2024). For many years, derogation of divergent ideological groups and values were understood as a primary right-wing phenomenon. From this *ideological asymmetry* perspective, right-wingers have been characterized as cognitively rigid and threat-averse (Jost et al., 2003) with an active preference for hierarchical structures that lead to heightened derogation of ideological others. More recently, however, *ideological symmetry* perspectives have challenged this account, with research demonstrating that left-wingers derogate right-wingers (Crawford & Brandt, 2020) and divergent ideological views (Frimer et al., 2017) to an equivalent extent. To explain this finding, it has been proposed that different kinds of left-wingers may derogate out-groups to groups to a greater or lesser extent.

Building on political science (Lilla, 2018) and ideological survey data (Hawkins et al., 2018), the Progressive Values Scale (PVS; Proulx et al., 2023) distinguishes between two politically active left-wing identities: *Progressives* who emphasize personal identity, activist expression and harm avoidance, and *Traditional Liberals* who emphasize consensus politics, gradual institutional change and free expression. Proulx et al. (2023) found both left-wing identity kinds generated unflattering visual representations of (out-group) Conservatives and flattering visual representations of (in-group) Liberals. However, Progressives generated especially positive images of fellow Liberals, and especially negative images of out-group Conservatives, suggesting a heightened propensity of Progressives (vs. Traditional Liberals) to derogate ideological others. Here, we build upon these findings by examining whether Progressives are also particularly negative in their appraisal of a fellow left-wing figure holding a dissenting view, rather than a broadly out-group right-wing target.

In recent years, author J.K. Rowling (Harry Potter book series) has been a culturally salient left-wing dissenter, expressing ideologically unaligned “gender critical” (vs. “self-identification”) views across social media platforms. Across two studies, we tested whether Progressives generate especially unflattering images of Rowling, relative to those generated by Traditional Liberals. In Study 1, we used reverse correlation methods (Dotsch & Todorov, 2012) to determine whether UK left-wing students generate differentially negative images of Rowling based on their Progressive or Traditional Liberal leanings. In Study 2, we sought to replicate and extend these findings with US adults, comparing depictions of Rowling (concrete exemplar) with a generalized example of someone holding the same “gender critical” views (abstract exemplar), as well as concrete (Lady Gaga) and abstract exemplars of those holding ideologically aligned “self-identification” beliefs. Our overarching aim was to provide the first evidence that the divergent propensity toward derogation *within* the left-wing also applies to a target from *within* the left who holds dissenting views.

### Mutually Derogating Depictions Between Left and Right

In contrast to evidence of ideological asymmetry detailing predominantly right-wing out-group derogation (for a review, see Jost, 2017), evidence for symmetrical derogation has been demonstrated with respect to the targeted derogation of directly ideologically opposing others and opinions (e.g., Crawford, 2014). While this research typically draws on self-reported, explicit attitudes toward targets, researchers have begun to employ multi-dimensional, data-driven methods of assessing divergent qualities of out-group derogation (e.g., relational responding task; De Houwer et al., 2015, automated text analysis; Markowitz, 2023). One such method is reverse correlation (Brinkman et al., 2017), a paradigm that incorporates two stages. First, a sample of participants (i.e., generators) is presented with two versions of a neutral base-face image, distinguished only by reversed patterns of random visual noise. Generators select which image best represents a target category (e.g., atheists; Brown-Iannuzzi et al., 2018). Over an aggregated series of trials, averaged over a sample of generators, a single classification image is derived representing consensual perceptions of the target category. Next, a subsequent sample (i.e., raters), unaware of how the faces were generated, evaluates the image, usually alongside its opposite, on a series of dimensions (e.g., attractiveness, intelligence). It has been argued that this double-blind implicit method can capture biases in a manner that avoids many criticisms of the IAT (Brinkman et al., 2017) and does so in a manner that allows for the emergence of qualities that could not have been apparent with explicit self-report judgments (Allbutt et al., 2008). Toward this end, Petsko and Kteily (2024) found that while both Conservatives and Liberals generated images of one another that were judged to be unflattering, they did so in unique and surprising ways, with the authors noting that Conservatives represented Liberals with immature faces, and Liberals represented Conservatives with savage faces. While this study provides a strong example of symmetrical inclinations to “dehumanize” one another, it may also be the case that different *kinds* of left-wingers may have distinctively influenced this aggregate “Liberal” depiction of Conservatives.

### Divisions Within the Left

For this to be the case, there would need to be identifiable kinds of left-wingers and a means to empirically identify them. However, while political and social psychologists have developed measures of ideological *extremity* within the Left (e.g., Left-wing Authoritarianism, Conway et al., 2018; Costello et al., 2022), assessments of left-wing *kinds* have not been as forthcoming when compared to other social sciences. For example, following the 2016 US election, political scientist Mark Lilla (2018) attributed Donald Trump’s victory to a divided left-wing, resulting from a division between a *classical* liberalism predicated on broad class coalitions and voter persuasion, and an emergent *identity* liberalism, portrayed as an activist focus by special interest groups. At the same time, the More in Common Foundation (MiC; Hawkins et al., 2018) published its landmark *Hidden Tribes* report, with findings suggestive of distinct tribes within the typically construed left/right divide. Within the left, the report differentiated *Traditional Liberals*—described as older, better off, satisfied with the political system and consensus-focused—from *Progressive Activists*—described as younger, urban, identity-focused, and angry. Subsequent work by the MiC identified analogous left-wing splits within the UK (Juan-Torres et al., 2020), France (Demoures et al., 2020), and Germany (Krause & Gagné, 2019).

Within the political and social psychology literature, the only measure developed to distinguish between these left-wing kinds, to our knowledge, is the Progressive Values Scale (PVS; Proulx et al., 2023). This measure distinguishes Progressive and Traditional Liberal views across four factors: (a) relative support for *Mandated Diversity* initiatives in institutional settings, (b) relative preferences for a *Recourse to Existing Institutions* relative to activist strategies in bringing about social equality, (c) relative concerns about *Cultural Appropriation* of marginalized groups by socially dominant groups, and (d) relative preferences for *Public Censure* of the expression of views deemed morally objectional, especially in the context of social media. Proulx et al. (2023) found PVS scores predicted divergent views on a variety of relevant issues (e.g., Progressive voting preference for US Senator Bernie Sanders over US President Joe Biden, Traditional Liberal preferences for neo-liberal economic policies). Further, Progressives were relatively likely to derogate Conservatives in cognitive domains, finding them to be less evolved and less understanding, relative to judgments made by Traditional Liberals. Of particular relevance, Proulx et al. (2023) used reverse correlation, having Progressives and Traditional Liberals generate classification images of Conservatives and Liberals. Here, the Conservative face generated by Progressives (vs. Traditional Liberals) was judged as relatively less intelligent and evolved (see Figure 1).

**Figure 1.**
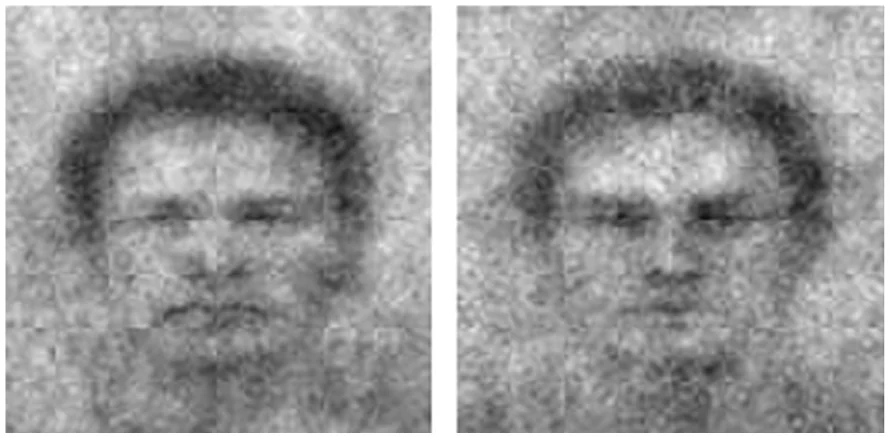
Classification Images of “Conservatives” Generated by (Left to Right) Progressives and Traditional Liberals (Proulx et al., 2023).

Since Trump’s first presidential victory, right-wing populists have achieved parliamentary wins across Western Europe (e.g., “Party for Freedom” in the Netherlands, the “Alternative for Deutschland” in Germany). At the same time, discussions of a divided political left-wing have become more frequent and more pointed among social scientists and cultural commentators across the political spectrum. In the book *Troubled*, Henderson (2025) distinguishes and characterizes progressive left-wingers in terms of expressed *luxury beliefs* intended to signal higher education and elite social status. Al-Gharbi (2024) offers a similar critique from within the left-wing, arguing that the social justice activism that characterizes progressives, however sincere, ultimately perpetuates their privileged social status. Recently, *Abundance* (Klein & Thompson, 2025) has been offered as an alternative left-wing equality agenda to progressive left-wing strategies, more in keeping with pre-identity liberalism (Lilla, 2018). With the re-election of Trump in 2024, cultural commentators have argued that an *abundance agenda* has formalized a “civil war” within the US left-wing, characterizing this perspective as focusing on consensus liberalism, in contrast with identity liberal activism. For example, while they may share views with identity liberals on a variety of broadly divisive social issues, they will avoid taking public stances on these issues, or otherwise calling out others over rights issues associated with identity classes e.g., debates over “the participation of trans women in college sports” (Chait, 2025). Indeed, a prominent issue over which identity liberals are likely to call out those with dissenting views is gender identity—specifically, how one warrants the belief that they can be considered men, women, neither or both.

### Gender Identity and In-Group Dissent

Debates on gender-based issues such as trans women in sports and age of transition (Tryl et al., 2022) have only grown in prevalence and intensity over the past decade, with an enhanced societal focus on gender identity across the political spectrum (Montiel-McCann, 2023). Stances on these issues predominantly align with a *gender self-identification* perspective, wherein individuals should be able to self-determine whether they are a man or a woman, regardless of biological sex at birth (e.g., “trans women are women,” Dawson, 2020; Sharpe, 2020) or a *gender critical* perspective, wherein biological sex maintains a distinct and paramount role in determining whether someone is a man or woman (e.g., “being a woman is a biological fact, not a feeling,” Gilligan, 2019, para. 4). While most left-wingers support *self-identification* (Pew Research Center, 2022; Smith, 2022), several culturally prominent left-wingers have dissented from this modal view—most notably, popular fantasy writer J.K. Rowling, author of the *Harry Potter* series.

A decade ago, Rowling was positively regarded among the Left through her role as a donor to the UK left-wing Labor Party (The Guardian, 2008) and global support for numerous women’s rights charities (Hills, 2020), abortion access (Frost, 2017), and marriage equality legalization (Waxman, 2015). Since then, Rowling has gone from “an unobjectionable matron of the political left to one of its most hated villains,” through her expression of gender critical views (Wheaton, 2022, para. 6), including social media posts expressing concerns about the sharing of single-sex spaces and legislation allowing people to legally change their gender without medical evidence (Romano, 2024). Within the Left, responses to this position were mixed. Some left-wing celebrities condemned Rowling’s actions and distanced themselves from any association with her (Noor, 2019). Essays were published with more than a thousand authors signing their disagreement with her gender critical stance (Flood, 2020). Other reactions within the Left were more favorable, with some authors disagreeing that Rowling’s gender identity stance constituted “hate speech” (Greenhalgh, 2020). Approached from the in-group dissent literature, the anger around Rowling’s views among fellow left-wingers can be explained in terms of key features of Rowling’s dissent. Broadly speaking, an in-group member violating a norm threatens group cohesion, motivating efforts to punish the dissenter (Jetten & Hornsey, 2014). Evaluations of an in-group dissenter are more negative when they comment publicly (Hornsey et al., 2005), show no remorse (Carlsmith et al., 2002) and are unwilling to change their stance (Chan et al., 2010), all features of Rowling’s public expression of her gender critical views. Nevertheless, it remains to be determined whether those who are relatively Progressive (vs. Traditionally Liberal) within the Left are less tolerant of these dissenting beliefs—mirroring the heightened derogation of ideological out-group Conservatives expressed by Progressives (Proulx et al., 2023).

### The Present Research

Over two experiments, we tested whether, within a left-wing sample, Progressives would more harshly derogate an in-group dissenter than Traditional Liberals, in keeping with Progressives’ greater derogation of an out-group target. Specifically, we expected that Progressives would generate a more negative image of an in-group dissenting left-winger holding gender critical views. In Study 1, Progressives and Traditional Liberals generated their visual representation of Rowling, in the same reverse correlation procedure used in Proulx et al. (2023). Next, a different sample of raters evaluated the J.K. Rowling face generated by each group, naïve to its origin. We expected the Progressive representation of J.K. Rowling to be evaluated more negatively (H1). Study 2 aimed to replicate these effects and determine whether Progressives’ enhanced biases applied to left-wing dissenters more generally, beyond the example of J.K. Rowling. Here, Progressives and Traditional Liberals generated a visual representation of a fellow left-winger who expressed either gender critical *or* self-identification views, described either in abstract (e.g., “*proponents of self-identification believe . . .*”) or concrete terms (e.g., Lady Gaga, who maintains *self-identification* views). We expected that overall, gender critical faces would be more negatively evaluated than self-identification faces (H2) and that this would especially be true for Progressives’ (relative to Traditional Liberals’) depictions (H3). Regarding the Progressive-generated gender critical faces, we expected the concrete exemplar (J.K. Rowling) to be rated most negatively, due to the increased salience of factors such as sustained public expression as presented in the in-group dissent literature (H4).

## Study 1

As an initial test of H1, a sample of left-wing UK university students generated their mental representation of J.K Rowling. Two classification images were generated, one by Progressives, a second by Traditional Liberals. Subsequently, these images were evaluated by a separate sample, who were naïve to how the images were generated. Raters evaluated both images on a range of attributes (e.g., intelligence, warm, competent, attractive, feminine, left-wing, moral).

### Method

Note: The materials, data and codes for both studies are available at (https://osf.io/z9kha/).

#### Image Generation Phase

##### Participants

135 Cardiff University students participated in this phase of the experiment. As we were interested in left-wingers, we only included participants who self-identified as left-wing (below the midpoint on a 1 = *Left-Wing* to 7 = *Right-wing)* scale. After this exclusion, 94 participants were left—a sufficient sample size per classification condition (Petsko & Kteily, 2024; Proulx et al., 2023). Out of these, eight participants’ data was missing and four were excluded due to their score being equal to the median, leaving 82 left-wing participants (81.70% female, 15.90% male, 2.40 % non-binary; *M_age_* = 20.07, SD_age_ = 2.18; 87.80% White, 4.90% Asian, 3.70% Mixed, 2.40% Black and 1.20% Other). Participants received course credit for their involvement. The study was approved by Cardiff University’s School of Psychology Ethics’ Committee.

##### Materials and Procedure

This study was not pre-registered. Participants completed the PVS (Proulx et al., 2023) in Qualtrics and were then presented with several media headlines framing the debate around J.K. Rowling’s stance on gender identity (e.g., “J.K. Rowling Comes Out As A TERF”; Ennis, 2021) and social media interactions where Rowling expresses gender critical views (e.g., “‘People who menstruate.’ I’m sure there used to be a word for those people . . .”; see Study 1 Materials (Generation) on OSF – https://osf.io/z9kha/files/osfstorage). Participants then began the reverse correlation task in Pavlovia, which presented 400 pairs of a base female face (a composite female face from the Karolinska Institute database; Lundqvist & Litton, 1998). While previous research examining mental representations of public figures has used genuine images as base faces (e.g., Democrat vs. Republican representations of Mitt Romney, Young et al., 2014), this may have minimized the opportunity for participants to project their relevant biases. As such, we selected a neutral composite female base face (i.e., a blank slate). For each image pair, one face was superimposed with a random white noise pattern, while the other was superimposed with the opposite noise pattern, as generated by the R package rcicr (Dotsch & Todorov, 2012). In each trial pair presentation, participants were asked to select the image of “*Who best resembles your mental representation of J.K. Rowling?*” There was a break after every 100 trials.

To ensure that participants understood J.K. Rowling as someone who endorsed gender critical views, upon completing the reverse correlation task participants were asked, “*Which of the following descriptions do you think best describes J.K. Rowling’s stance on gender identity?*” These following descriptors matched the definitions provided in the Study 1 Materials (Generation) available on OSF (see https://osf.io/z9kha/files/osfstorage), along with an additional descriptor for ambivalence (“*Some people are ambivalent about gender identity, maintaining beliefs associated with both gender self-identification and gender critical perspectives. For example, their opinion on whether trans women are women is mixed*”). A total of 75.30% of participants correctly identified J.K. Rowling’s view as gender critical, 17.30% as ambivalent and 7.40% as in favor of self-identification.

Following the procedure of Proulx et al. (2023), PVS responses were divided using a median split (*Mdn* = 4.50), distinguishing Traditional Liberals (low on the PVS) and Progressives (high on the PVS). A classification image was created for each group, using the rcicr package. The two images (see Figure 2) were subsequently evaluated in the rating phase.

**Figure 2.**
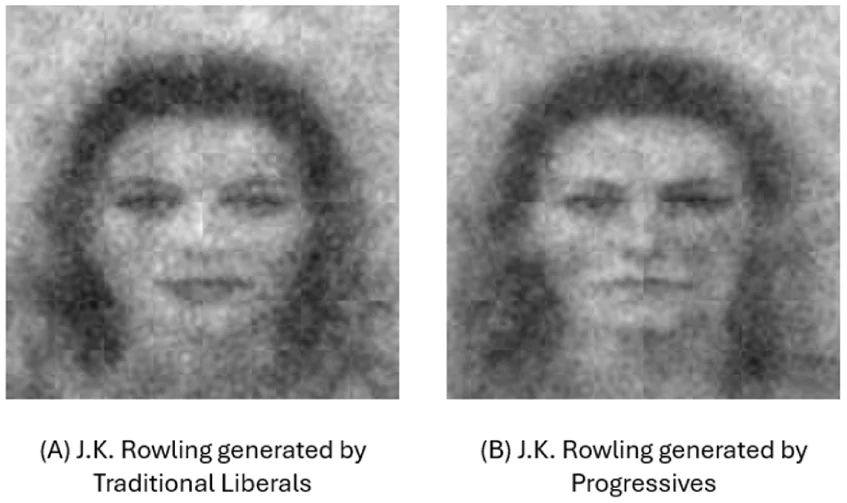
Classification Images Generated by Traditional Liberals and Progressives of J.K. Rowling.

#### Image Rating Phase

##### Participants

A total of 178 Cardiff University students (*M_age_* = 19.23, *SD_age_* = 1.34) participated for course credit. The majority were women (80.90% female, 14% male, 3.40% non-binary, 1.10% transgender, .60% other) and White (72.50% White, 15.70% Asian, 5% Other, 3.40% Mixed, 3.40% Black). All participants served as raters, regardless of their political orientation (measured 1 = *Left-Wing* to 7 = *Right-Wing*) or self-reported political category (*Conservative*, *Liberal*, *Libertarian*, *Progressive*). A sensitivity power analysis for the paired-sample *t*-test, conducted in G*Power (Faul et al., 2007; α = .05, power = .80) indicated that our sample size was sufficiently powerful to detect a minimum effect size of *f* = .211. A sensitivity power analysis for the one-sample *t*-test, conducted in G*Power (Faul et al., 2007; α = .05, power = .80) indicated that our sample size was sufficiently powerful to detect a minimum effect size of *f* = .212.

##### Materials and Procedure

Participants completed the survey on Qualtrics. They rated the two classification images on 11 attributes (intelligence, likable , warm, competent, attractive, feminine, left-wing, moral, hard-working, young, unprejudiced toward trans people). All ratings were made on a seven-point scale (e.g., 1 = Cold, 7 = Warm; this was reversed from the original 1 = Warm, 7 = Cold). Many of the attributes have been used to compute indices of warmth and competence across reverse correlation studies (Brinkman et al., 2017; Han et al., 2023). Consistent with past research (e.g., Brown-Iannuzzi et al., 2018), we included an item assessing whether raters would apply similar attributions to the images as the generators, that is, whether the image was “prejudiced/unprejudiced toward trans people” (with lower scores pointing to seeing the face as prejudiced toward trans people). Finally, raters completed the PVS scale (Proulx et al., 2023). As an exploratory analysis, a sub-sample of left-wing participants (who selected 4 or below for the political orientation and either Liberal or Progressive for the political category) was selected to observe whether there would be any correlation between PVS and ratings of the two J.K. Rowling faces (see Supplementary Materials—Table S1.2).

### Results

#### Factor Analysis on Traits

Given the established status of warmth and competence as critical dimensions in social perception (Fiske et al., 2007), we carried out an EFA on the traits most associated with these dimensions (warm, likable, hard-working, competent, intelligent). We also included attractiveness, as this has been found to be linked with warmth (see Han et al., 2023). A principal axis factor analysis was conducted with oblique rotation. The Kaiser–Meyer–Olkin measure verified the sampling adequacy for the analysis (KMO = 0.77), and all KMO values for individual items were greater than 0.68, above the acceptable limit of 0.50 (Field, 2024, Hair et al., 2006). Two factors had Eigenvalues greater than one, explaining 72.02% of the variance. Table S1.1 (see Supplementary Materials S1) shows the factor loadings after rotation. The items that cluster on Factor 1 capture Warmth, while Factor 2 captures Competence.

#### Trait Analyses

As seen in Table 1, in line with our hypothesis, paired-sample *t*-tests revealed that the Progressive-generated J.K Rowling face was rated *as colder and less competent* as well as being rated less *left-wing, feminine, moral, young* and *prejudiced toward trans people* than the Traditional Liberal–generated face (for the corresponding bootstrapped coupled estimation effect sizes see Supplementary Materials—S2, Figure S2). Furthermore, when compared to the scale midpoint (4), one-sample *t*-tests, which were Holm (1979) corrected, revealed that the Progressive-generated J.K Rowling face was seen as *cold, unattractive, feminine, right-wing, immoral, old* and *prejudiced against trans people*. In contrast, the Traditional Liberal–generated J.K. Rowling faces was seen as *warm*, *competent, feminine, moral, left-wing, young* and *unprejudiced toward trans people*.

**Table 1.** Mean Ratings, Paired Sample and One-Sample t-Test Values for the Warmth, Competence and the 5 Other Traits per Generator Type for the J.K Rowling Face Generation.

Trait	Generator	Comparison			Trait Expression	
		*M* [95% CI]	*t*	Cohen’s *d* [95% CI]	One sample *t* (vs. 4)	Cohen’s *d* [95% CI]
Warmth	TL	4.25 [4.10, 4.40]	16.04***	1.20 [1.01, .1,39]	3.25***	.25 [.10, .40]
	P	2.85 [2.71, 2.99]			–15.97***	–1.20 [–1.39, –1.00]
Competence	TL	4.55 [4.40, 4.69]	5.86***	.44 [.28, .59]	7.36***	.55 [.40, .71]
	P	4.00 [3.84, 4.16]			–.02	–.00 [–.16, .14]
Moral	TL	4.44 [4.27, 4.60]	7.93***	.59 [.43, .75]	5.20***	.39 [.24, .54]
	P	3.64 [3.46, 3.82]			–4.01***	–.30 [–.45, –.15]
Unprejudiced	TL	4.47 [4.29, 4.66]	10.35***	.77 [.61, .94]	5.02***	.38 [.22, .53]
	P	3.28 [3.09, 3.47]			–7.63***	–.57 [–.73, –.41]
Feminine	TL	5.66 [5.51, 5.82]	8.48***	.64 [.47, .80]	20.88***	1.57 [1.34, 1.78]
	P	4.81 [4.62, 5.01]			8.12***	.60 [.44, .76]
Left-Wing	TL	4.36 [4.18, 4.54]	6.86***	.51 [.36, .68]	3.92***	.29 [.14, .44]
	P	3.56 [3.36, 3.75]			–4.50***	–.34 [–.49, –.18]
Young	TL	4.44 [4.26, 4.62]	11.58***	.87 [.69, 1.04]	4.79***	.36 [.21, .51]
	P	3.24 [3.07, 3.42]			–8.54***	–.64 [–.80, .48]

*Note.* TL = generated by Traditional Liberals; *P* = generated by Progressives; CI = confidence interval.

* *p <* .05. **p < .005. ***p < .001.

#### Discussion

Overall, we found strong support for our hypothesis: A Progressive-generated face of a fellow left-winger expressing gender critical views (J.K. Rowling) elicited negative evaluations, in both relative and absolute respects. Across an array of attributes, spanning qualities relevant to warmth and competence, as well as morality and prejudice, the Progressive-generated face was judged more negatively, along with being judged as relatively less feminine, less youthful and relatively right-wing. With the exception of the competence and feminine judgments, this was also true in absolute terms, where the Progressive-generated face was seen as actively lacking the judged qualities. More striking was a pattern of effects that we did not anticipate: The Rowling face generated by Traditional Liberals was a face-validly flattering depiction and was judged accordingly by naïve raters. In absolute terms, the Traditional Liberal face was judged to be warm, competent, moral, lacking in prejudice, as well as feminine, youthful and left-wing. As such, the Rowling images generated by Progressive and Traditional Liberals did not merely differ in (negative) *degree*, but in valenced *kind*, as actively and broadly negative and positive images. These findings offer a more striking pattern of results than the relatively negative depiction of Conservatives generated by Progressives in Proulx et al. (2023), which differed from Traditional Liberal faces only by degree of derogation. This offers substantial empirical support for the argument that these left-wing identities differ in kind, rather than degree.

While we expected Progressives to derogate an in-group dissenter more than Traditional Liberals, we were surprised that the latter engaged in no derogation whatsoever of a gender critical advocate. In Study 2, we aimed to differentiate the propensity of these left-wing kinds to derogate a gender critical in-group dissenter in the *abstract* (i.e., by definition) from judgments of J.K. Rowling as a *concrete* exemplar. While Traditional Liberals may hold positive or mitigating views of Rowling relative to Progressives, it may nevertheless be possible that they derogate an unspecified other holding gender critical views to an equivalent extent to Progressives.

## Study 2

Study 2 was designed to replicate and extend our findings in two crucial respects. First, we added a condition where a sub-sample of generators provided their representation of an unspecified individual who expressed gender critical beliefs (*abstract*), with the aim of determining whether the divergent depictions generated by Progressives and Traditional Liberals were specific to differentially salient features of Rowling (c*oncrete*) or represent perceptions of gender critical left-wingers more generally. Second, we added a condition where some generators provided their representation of a fellow left-winger holding a *self-identification* view, in either an *abstract* (definition) or *concrete* (Lady Gaga) manner. This allowed us to test whether Progressives and Traditional Liberals meaningfully distinguish between those in favor of self-identification versus holding gender critical beliefs. We also emphasized the ideological in-group status of both targets (i.e., described as “fellow left-wingers”). Finally, we aimed to replicate and extend our findings beyond UK university students, sampling a population of left-wing US adults.

Study 1 reaffirmed that self-identification views predominate among self-identified left-wingers. We therefore expected the dissenting gender critical faces to be rated more negatively than the self-identification faces (H2). This would be represented by a significant main effect of gender identity stance. Further, we expected the gender critical faces generated by Progressives to be rated more negatively than the gender critical faces generated by Traditional Liberals (H3). This would be represented by a significant interaction between generator type and gender stance. Finally, as noted in our Study 2 pre-registration https://osf.io/25kc7 (which included the planned sample size, study design, exclusion criteria and planned primary analyses), we expected that among Progressive generators, the concrete dissenter face would be evaluated more negatively than the abstract face (H4), based on the increased salience of characteristics that heighten derogation (i.e., a public, unapologetic and unwavering in-group dissent).

### Method

#### Image Generation Phase

##### Participants

We recruited 532 American adults via Prolific, who participated in exchange for approximately £6–7/hr. Following Proulx et al. (2023), to ensure a broad left-wing sample we limited our final sample to those identifying as “left-wing” or “moderate,” removing those who identified as relatively conservative (5–7 on a 7-point Left-wing to Right-wing scale), and selecting only those who then identified as “Progressive” or “Liberal” (vs. “Libertarian” or “Conservative”). This left us with 432 participants. We also removed nine participants whose data were missing, as well as excluding participants who failed two or more of the four attention checks introduced in Study 2’s reverse correlation task and those whose PVS score was equal to the median. This resulted in a final sample size of 382 (*M_age_* = 39.70, SD_age_ = 13.82; 58.40% female 36.90% male, 3.70% non-binary, 1% transgender). Most participants were White (62.80% White, 13.90% African American, 9.90% Asian, 8.90% Latino, 3.90% Other, 0.50% Native American). 53.10% were educated to a higher level (e.g., undergraduate and above), 48.20% were in full-time employment. The study was approved by Cardiff University’s School of Psychology Ethics Committee.

##### Materials and Procedure

After completing the PVS (Proulx et al., 2023), generators were randomly assigned in Qualtrics to one of four conditions where they were asked to imagine “a *fellow left-winger*” with one of two stances on gender identity. In the abstract conditions, participants read a brief description of what a left-winger in favor of self-identification versus gender critical would believe (see also Study 2 Materials (Generation) available on OSF (https://osf.io/z9kha/files/osfstorage). In the self-identification condition, participants were told that “*Proponents of GENDER SELF-IDENTIFICATION believe that a person should be allowed to identify as their chosen gender, regardless of their biological sex at birth. For example, they would agree that ‘trans women are women.*” In the gender critical condition, participants were told that “*Proponents of GENDER CRITICAL beliefs believe that biological sex primarily determines whether someone is a man or a woman, rather than a chosen gender. For example, they would agree that ‘trans women are not women in the same way as women who were born female.*”

In the concrete conditions, participants were given an example of a public left-wing figure who was either in favor of a self-identification or gender critical stance, accompanied by a brief introduction outlining the topic of gender identity and followed by social media posts illustrating the example’s respective gender identity position (see Study 2 Materials Generation available on OSF – https://osf.io/z9kha/files/osfstorage). As with Study 1, we used J.K. Rowling as a concrete example of someone espousing gender critical beliefs. For the self-identification concrete condition, we selected Lady Gaga, a well-known public figure and strong advocate of self-identification within the LGBTQ+ community (Welsh, 2024). As a manipulation check, we asked participants to identify the gender stance of the abstract description and the concrete exemplar. 94.60% correctly matched the self-identification description with its label, and 92% correctly matched the gender critical description with its label. Meanwhile, 100% correctly identified Lady Gaga’s stance as self-identification, and 94.30% correctly identified J.K. Rowling’s stance as gender critical.

The generation task consisted of 404 trials (four of which were attention check trials not used for classification image generation) in Pavlovia. Depending on the assigned condition, the instructions were as follows: (Abstract vs. Concrete—*Who best resembles a fellow left-winger who believes in gender self-identification/is gender critical?* vs. *Who best resembles your mental representation of fellow left-winger Lady Gaga/J.K. Rowling?*). There was a break after every 101 trials. Study 2 included four attention checks, where the stimulus pair was an image of an adult or a child superimposed (child image stimuli were taken from Study 10A of Wolf et al., 2024) with white noise, with participants asked to select the adult image.

As in Study 1, participants were grouped into Progressive (*M*_age_ = 37.85; 65.40% female, 27.60% male) or Traditional Liberal (*M*_age_ = 41.43; 51.80% female, 45.70% male) generators via a median split (*Mdn* = 4.36) on the PVS. This allowed for the generation of eight classification images (see Figure 3). Participants also indicated what stance they aligned with, self-identification or gender critical, as a way of demonstrating that belief in self-identification is the modal position among our left-wing cohort. Overall, 63.90% of participants were in favor of self-identification, and 36.10% were gender critical. Finally, depending on the condition they were assigned to, participants were asked two exploratory questions regarding someone who believes in self-identification/is gender critical/Lady Gaga or J.K. Rowling: (e.g., 1. *To what extent do you have positive or negative feelings toward someone who is in favor of gender self-identification/is gender critical/Lady Gaga or J.K. Rowling?”*; 2. *To what extent do you believe someone who is in favor of gender self-identification/is gender critical/Lady Gaga or J.K. Rowling to be a positive influence on society?*) on a 7-point scale (1 = Very Negative, 7 = Very Positive). The results for these analyses are included in Supplementary Materials—Table S3.

**Figure 3.**
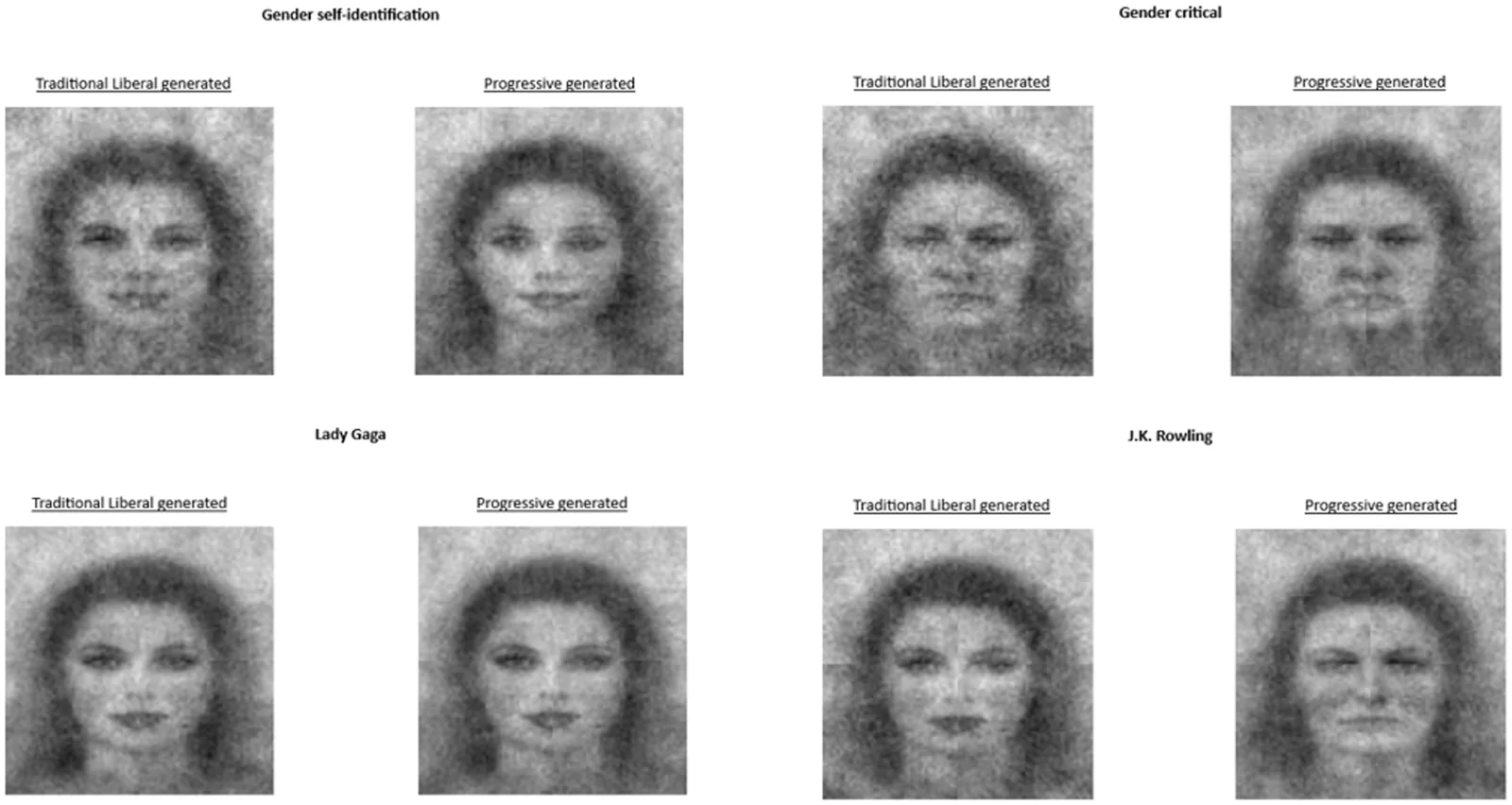
First Row Contains (From Left to Right) for the Abstract Condition, Traditional Liberal–Generated Self-Identification Face, Progressive-Generated Self-Identification Face, Traditional Liberal–Generated Gender Critical Face and the Progressive-Generated Gender Critical Face. *Note*. The second row follows the same order but for concrete instructions (i.e., Lady Gaga, J.K. Rowling).

#### Image Rating Phase

##### Participants

A total of 311 US Prolific users (*M*_age_ = 37.96, *SD*_age_ = 14.24) served as raters and were paid approximately £6–7/hr. After removing 10 incomplete responses, we were left with 301 participants. In all, 56.80% self-identified as female, 42.20% self-identified as male, and 1% as non-binary/transgender. A total of 52.50% participants were White, 16.60% African American, 18.30% were Asian, 10.3% were Latino, 1.30% Other, and 1% Native American. An a priori power analysis conducted using G*Power (Faul et al., 2007) using a small to medium effect size (*f* = .18) and an alpha of .05 showed that a total sample of 245 participants was required to achieve a power of .80 for a three-way repeated measures ANOVA. We collected data from 311 participants to account for any potential data loss. A sensitivity power analysis for the one-sample t-test, conducted in G*Power (Faul et al., 2007; α = .05, power = .80) indicated that, with our sample size, the study was sufficiently powerful to detect a minimum effect size of *f* = .161.

##### Materials and Procedure

Raters completed the survey on Qualtrics. They completed demographic questions, including their political orientation (1 = *Left-Wing*, 7 = *Right-Wing*) and the political category they identified with (*Conservative*, *Liberal*, *Libertarian*, *Progressive*). Next, participants rated all eight images on 12 attributes (*intelligence, likable , warm, competent, attractive, feminine, left-wing, moral, hard-working, young, unprejudiced toward trans people, belief in self-identification*). Faces and attributes were presented in a random order.

All ratings were made on a seven-point scale (e.g., 1 = *Cold*, 7 = *Warm*; this was reversed from the original 1 = *Warm*, 7 = *Cold*). Finally, they completed the PVS (Proulx et al., 2023). As an exploratory analysis, a sub-sample of left-wing participants (who selected 4 or below for the Political Orientation and either *Liberal* or *Progressive* for the political category) was selected to observe whether there would be any correlation between PVS and ratings of the eight prototype faces (see Supplementary Materials—Table S4.2).

### Results

#### FA on Traits

As with Study 1, a principal axis factor analysis was conducted on the same six traits corresponding to the Warmth and Competence dimensions, with oblique rotation (direct oblimin). The Kaiser–Meyer–Olkin measure verified the sampling adequacy for the analysis (KMO = 0.86), and all KMO values for individual items were greater than 0.83, above the acceptable limit of 0.50 (Field, 2024). Two factors had Eigenvalues greater than 1, explaining 80.02% of the variance. Table S4.1 (see Supplementary Materials S4) shows the factor loadings after rotation. Factor 1 captures a Warmth dimension (α = .90), while Factor 2 represents Competence (α = .81).

#### The Effect of Political Identity, Gender Stance, and Information Type on Participant Evaluations

As noted, three primary effects were examined (see Table 2). First, we were interested in the main effect of gender identity stance on participant ratings (H2). Second, we focused on the two-way interaction between generator type (i.e., political identity of generator: Progressive or Traditional Liberal) and gender identity stance on participants’ evaluations (H3; see Table 3 and for the equivalent bootstrapped coupled estimation effect sizes – Ho et al., 2019 – see Figure S5 in Supplementary Materials S5). Finally, we investigated whether the Progressive-generated concrete gender critical face (J.K. Rowling) would be rated more negatively than the Progressive-generated abstract gender critical face (H4). Our descriptions that follow focus on the three effects most relevant to our hypotheses; however, all effects are presented in Table 4 (for the equivalent bootstrapped coupled estimation effect sizes see Table S5.1 in Supplementary Materials S5).

**Table 2.** Main Effects, Two-Way, Three-Way Interactions, and Effect Sizes 95% CI for the Repeated Measures ANOVA on Ratings for Valenced and Non-Valenced Traits.

	Valenced Traits								Non-valenced Traits							
	Warmth		Competence		Moral		Unprejudiced toward trans people		Feminine		Left-Wing		Young		Belief in self-id	
Primary Effects	*F*	Cohen’s *f* [95% CI]	*F*	Cohen’s *f* [95% CI]	*F*	Cohen’s *f* [95% CI]	*F*	Cohen’s *f* [95% CI]	*F*	Cohen’s *f* [95% CI]	*F*	Cohen’s *f* [95% CI]	*F*	Cohen’s *f* [95% CI]	*F*	Cohen’s *f* [95% CI]
**GT**	36.62***	.34 [.23, .46]	13.06***	.20 [.09, .32]	1.20	.02 [0, .18]	9.64**	.17 [.06, .29]	116.22***	.62 [.50, .74]	6.38*	.13 [.03, .26]	23.12***	.27 [.16, .39]	11.87***	.19 [.08, .31]
**GIS**	814.48***	1.64 [1.47, 1.82]	195.41***	.80 [.67, .93]	157.03***	.72 [.59, .85]	239.11***	.89 [.76, 1.02]	677.57***	1.50 [1.33, 1.66]	135.97***	.67 [.55, .80]	882.95***	1.71 [1.53,1.89]	209.17***	.83 [.70, .96]
**IT**	400.59***	1.15 [1.01, 1.30]	117.42***	.62 [.50, .75]	25.24***	.28 [.17, .40]	58.21***	.43 [.32, .56]	634.56***	1.45 [1.29, 1.61]	33.83***	.33 [.22, .45]	298.36***	.99 [.86, .1.13]	57.23***	.43 [.32, .55]
GT * GIS	371.38***	1.11 [.97, 1.25]	136.95***	.67 [.55, .80]	60.32***	.44 [.33, .56]	87.06***	.53 [.42, .66]	478.37***	1.26 [1.11, 1.41]	65.59***	.46 [.35, .58]	461.77***	1.24 [1.09,1.39]	75.07***	.50 [.38, .62]
GT * IT	231.34***	.87 [.74, 1.01]	37.95***	.35 [.24, .47]	6.98*	.14 [.04, .26]	41.55***	.37 [.25, .49]	15.22***	.22 [.11, .34]	15.51***	.22 [.11, .34]	324.92***	1.04 [.90, 1.18]	47.99***	.39 [.28, .52]
GIS*IT	207.84***	.83 [.70, .96]	72.74***	.49 [.37, .61]	57.97***	.43 [.32, .56]	72.79***	.49 [.37, .61]	184.45***	.78 [.65, .91]	35.03***	.34 [.22, .46]	81.52***	.52 [.40, .64]	61.84***	.45 [.33, .57]
GT * GIS * IT	4.49*	.11 [0, .23]	.16	0 [0, .13]	1.18	.02 [0, .17]	.39	0 [0, .15]	32.65***	.32 [.21, .44]	.90	0 [0, .17]	2.62	.07 [0, .21]	.98	0 [0, .17]

*Note.* GT = generator type; GIS = gender identity stance; IT = information type; CI = confidence interval.

* *p* < .05. **p < .005. ***p < .001.

**Table 3.** Means, 95% CI, and p-Values for the Simple Pairwise Comparisons of the Interaction Between Gender Identity Stance and Generator Type.

Traits	Generator	Self-identification		Gender critical	
		*M*[95% CI]	*p*	*M* [95% CI]	*p*
Warmth	TL	4.77 [4.68, 4.87]	<.001	3.75 [3.67, 3.84]	<.001
	P	5.24 [5.14, 5.33]		2.95 [2.84, 3.06]	
Competence	TL	4.44 [4.37, 4.52]	<.001	4.12 [4.04, 4.20]	<.001
	P	4.67 [4.58, 4.76]		3.72 [3.62, 3.81]	
Moral	TL	4.41 [4.32, 4.50]	<.001	4.00 [3.92, 4.08]	<.001
	P	4.61 [4.51, 4.72]		3.72 [3.63, 3.81]	
Unprejudiced	TL	4.36 [4.27, 4.46]	<.001	3.71 [3.62, 3.80]	<.001
	P	4.58 [4.48, 4.69]		3.27 [3.17, 3.38]	
Feminine	TL	5.66 [5.55, 5.77]	<.001	4.97 [4.86, 5.09]	<.001
	P	6.10 [5.98, 6.21]		3.66 [3.52, 3.79]	
Left-wing	TL	4.20 [4.12, 4.28]	<.001	3.81 [3.72, 3.91]	<.001
	P	4.41 [4.32, 4.51]		3.44 [3.34, 3.54]	
Young	TL	4.99 [4.89, 5.10]	<.001	3.94 [3.85, 4.02]	<.001
	P	5.57 [5.47, 5.68]		3.02 [2.92, 3.13]	
Belief in self-id	TL	4.34 [4.25, 4.44]	<.001	3.68 [3.59, 3.78]	<.001
	P	4.51 [4.41, 4.62]		3.27 [3.16, 3.38]	

*Note.* TL = generated by Traditional Liberals; *P* = generated by Progressives.

**Table 4.** Means, 95% CI and p-Values for the Simple Pairwise Comparisons of the Interactions.

Trait	Self-identification					Gender Critical			
	Generator	Abstract		Lady Gaga		Abstract		J.K. Rowling	
		*M* [95%CI]	*p*	*M* [95%CI]	*p*	*M* [95%CI]	*p*	*M* [95%CI]	*p*
Warmth	TL	4.39^P^ [4.27, 4.51]	<.001	5.16^P^ [5.05, 5.26]	.578	2.86^N^ [2.73, 2.98]	.002	4.65^P^ [4.54, 4.76]	<.001
	P	5.34^P^ [5.23, 5.45]		5.14^P^ [5.03, 5.24]		2.66^N^ [2.53, 2.79]		3.23^N^ [3.12, 3.35]	
Competence	TL	4.28^P^ [4.18, 4.37]	<.001	4.61^P^ [4.51, 4.70]	.668	3.71^N^ [3.59, 3.82]	<.001	4.53^P^ [4.43, 4.62]	<.001
	P	4.72^P^ [4.62, 4.82]		4.62^P^ [4.52, 4.72]		3.50^N^ [3.37, 3.62]		3.94 [3.83, 4.04]	
Moral	TL	4.42^P^ [4.31, 4.53]	<.001	4.41^P^ [4.29, 4.52]	.008	3.71^N^ [3.58, 3.83]	.044	4.29^P^ [4.19, 4.40]	<.001
	P	4.69^P^ [4.56, 4.81]		4.54^P^ [4.43, 4.66]		3.56^N^ [3.43, 3.68]		3.88 [3.78, 3.99]	
Unprejudiced	TL	4.25^P^ [4.13, 4.37]	<.001	4.48^P^ [4.36, 4.60]	.862	3.25^N^ [3.11, 3.38]	.026	4.17^P^ [4.05, 4.29]	<.001
	P	4.70^P^ [4.57, 4.83]		4.47^P^ [4.35, 4.59]		3.08^N^ [2.94, 3.22]		3.46^N^ [3.34, 3.58]	
Feminine	TL	5.19^P^ [5.05, 5.33]	<.001	6.13^P^ [6.01, 6.25]	.331	4.15 [3.98, 4.32]	<.001	5.80^P^ [5.67, 5.93]	<.001
	P	6.02^P^ [5.89,6.15]		6.17^P^ [6.06, 6.29]		2.78^N^ [2.59, 2.97]		4.54^P^ [4.38, 4.70]	
Left-Wing	TL	4.12 [4.01,4.22]	<.001	4.28^P^ [4.17, 4.39]	.064	3.49^N^ [3.35, 3.63]	.021	4.14^P^ [4.04, 4.25]	<.001
	P	4.46^P^ [4.34, 4.58]		4.37^P^ [4.26, 4.48]		3.30^N^ [3.17, 3.44]		3.58^N^ [3.45, 3.70]	
Young	TL	4.47^P^ [4.33, 4.61]	<.001	5.52^P^ [5.40, 5.64]	.118	3.02^N^ [2.90, 3.14]	.118	4.85^P^ [4.73, 4.98]	<.001
	P	5.71^P^ [5.59, 5.83]		5.44^P^ [5.31, 5.56]		2.88^N^ [2.74, 3.02]		3.17^N^ [3.04, 3.30]	
Belief in self-id	TL	4.21^P^ [4.09, 4.33]	<.001	4.48^P^ [4.35, 4.60]	.316	3.22^N^ [3.08, 3.35]	.104	4.15 [4.02, 4.28]	<.001
	P	4.61^P^ [4.48, 4.73]		4.42^P^ [4.30, 4.54]		3.10^N^ [2.96, 3.25]		3.44^N^ [3.32, 3.57]	

*Note.* TL = generated by Traditional Liberals; *P (Generator)* = generated by Progressives; *P* = significantly different from the midpoint in a positive direction (above 4); *N* = significantly different from the midpoint in a negative direction (below 4).

##### Warmth

There was a significant main effect of gender identity stance, *F*(1, 299) = 814.48, *p* < .001, η^2^ = .73, *f* = 1.64. Overall, gender critical faces (*M* = 3.35) were judged as *colder* than those in favor of self-identification (*M* = 5.01). This supports H2. The two-way interaction between political identity of generator and gender identity stance was significant, *F*(1,299) = 371.38, *p* < .001, η^2^ = .55, *f* = 1.11. The Progressive-generated gender critical faces (*M* = 2.95) were rated as *colder* than the Traditional Liberal–generated faces (*M* = 3.75), *p <* .001. This supports H3. Conversely, for the self-identification stance, there was a significant effect in the opposite direction, where the Progressive-generated faces (*M* = 5.24) were rated as *warmer* than the Traditional Liberal–generated faces (*M* = 4.77), *p <* .001. Finally, comparing the Progressive-generated concrete and abstract gender critical faces, we found that the face generated from the concrete exemplar (*M* = 3.23) was rated as *warmer* than the face generated from the abstract exemplar (*M* = 2.66), *f =*.37, *p<*.001 (see also Table S5.2 in Supplementary Materials S5 for this and the other traits). This runs counter to H4.

##### Competence

There was a significant main effect of gender identity stance, *F*(1,299) = 195.41, *p* <. 001, η^2^ = .39, *f* = .80. Overall, gender critical faces (*M* = 3.92) were judged as less *competent* than those in favor of self-identification (*M* = 4.56). This supports H2. The two-way interaction between political identity of generator and gender identity stance was significant, *F*(1,299) = 136.95, *p* <. 001, η^2^ = .31, *f* = .67. The Progressive-generated gender critical faces (*M* = 3.72) were rated as less *competent* than the Traditional Liberal–generated faces, (*M* = 4.12), *p <* .001. This supports H3. Conversely, for the self-identification stance, there was a statistically significant effect in the opposite direction, where the Progressive-generated faces (*M* = 4.67) were rated as more *competent* than the Traditional Liberal–generated faces (*M* = 4.44), *p <* .001. Finally, comparing the Progressive-generated concrete and abstract gender critical faces, we found that the face generated from the concrete exemplar (*M* = 3.94) was rated as more *competent* than the face generated from the abstract exemplar (*M* = 3.50), *f* = .37, *p <* .001. This runs counter to H4.

#### Other Valenced Traits

##### Moral

There was a significant main effect of gender identity stance, *F*(1,299) = 157.03, *p* < .001, η^2^ = .34, *f* = .72. Overall, gender critical faces (*M* = 3.86) were judged as less *moral* than those in favor of self-identification (*M* = 4.51). This supports H2. The two-way interaction between political identity of generator and gender identity stance was significant, *F*(1,299) = 60.32, *p*<.001, η^2^ = .17, *f* = .44. The Progressive-generated gender critical faces (*M* = 3.72) were rated as less *moral* than the Traditional Liberal–generated faces (*M =* 4.00), *p <* .001. This supports H3. Conversely, for the self-identification stance, there was a statistically significant effect in the opposite direction, where the Progressive-generated faces (*M =* 4.61) were rated as more *moral* than the Traditional Liberal–generated faces (*M =* 4.41), *p <* .001. Finally, comparing the Progressive-generated concrete and abstract gender critical faces, we found that the face generated from the concrete exemplar (*M =* 3.88) was rated as more *moral* than the face generated from the abstract exemplar (*M =* 3.56), *f =*.25, *p <* .001. This runs counter to H4.

##### Unprejudiced Trans People

There was a significant main effect of gender identity stance, *F*(1,299) = 239.11, *p* < .001, η^2^ = .44, *f =*.89. Overall, gender critical faces (*M =* 3.49) were judged as less *unprejudiced toward trans people* than those in favor of self-identification (*M =* 4.47). This supports H2. The two-way interaction between political identity of generator and gender identity stance was significant, *F*(1,299) = 87.06, *p* < .001, η^2^ = .23, *f =*.53. The Progressive-generated gender critical faces (*M =* 3.27) were rated as less *unprejudiced toward trans people* than the Traditional Liberal–generated faces (*M =* 3.70), *p <* .001. This supports H3. Conversely, for the self-identification stance there was a statistically significant effect in the opposite direction, where the Progressive-generated faces (*M =* 4.58) were rated as more *unprejudiced toward trans people* than the Traditional Liberal–generated faces (*M* = 4.36), *p <* .001. Finally, comparing the Progressive-generated concrete and abstract gender critical faces, we found that the face generated from the concrete exemplar (*M =* 3.46) was rated as more *unprejudiced toward trans people* than the face generated from the abstract exemplar (*M =* 3.08), *f =*.27, *p <* .001. This runs counter to H4.

#### Non-Valenced Traits

##### Feminine

There was a significant main effect of gender identity stance, *F*(1, 299) = 677.57, *p* < .001, η^2^ = .69, *f =*1.50. Overall, gender critical faces (*M =* 4.32) were judged as less *feminine* than those in favor of self-identification (*M =* 5.88). The two-way interaction between political identity of generator and gender identity stance was significant, *F*(1,299) = 478.37, *p* < .001, η^2^ = .61, *f =*1.26. The Progressive-generated gender critical faces (*M* = 3.66) were rated as less *feminine* than the Traditional Liberal–generated faces (*M* = 4.97), *p <* .001. Conversely, for the self-identification stance there was a statistically significant effect in the opposite direction, where the Progressive-generated faces (*M* = 6.10) were rated as more *feminine* than the Traditional Liberal–generated faces (*M* = 5.66), *p <* .001. Finally, comparing the Progressive-generated concrete and abstract gender critical faces, we found that the face generated from the concrete exemplar (*M =* 4.54) was rated as more *feminine* than the face generated from the abstract exemplar (*M =* 2.78), *f =*.91, *p <* .001.

##### Left-Wing

There was a significant main effect of gender identity stance, *F*(1, 299) = 135.97, *p* < .001, η^2^ = .31, *f =*.67. Overall, gender critical faces (*M =* 3.63) were judged as less *left-wing* than those in favor of self-identification (*M =* 4.31). The two-way interaction between political identity of generator and gender identity stance was significant, *F*(1,299) = 65.59, *p* < .001, η^2^ = .18, *f =*.46 The Progressive-generated gender critical faces (*M* = 3.44) were rated as less *left-wing* than the Traditional Liberal–generated faces (*M* = 3.81), *p <* .001. Conversely, for the self-identification stance there was a statistically significant effect in the opposite direction, where the Progressive-generated faces (*M* = 4.41) were rated as more *left-wing* than the Traditional Liberal–generated faces, (*M* = 4.20), *p <* .001. Finally, comparing the Progressive-generated concrete and abstract gender critical faces, we found that the face generated from the concrete exemplar (*M =* 3.58) was rated more left-wing than the face generated from the abstract exemplar (*M =* 3.30), *f =*.17, *p <* .001.

##### Young

There was a significant main effect of gender identity stance, *F*(1, 299) = 882.95, *p* < .001, η^2^ = .75, *f =*1.71. Overall, gender critical faces (*M =* 3.48) were judged as *older* than those in favor of self-identification (*M =* 5.28). The two-way interaction between political identity of generator and gender identity stance was significant, *F*(1,299) = 461.77, *p* < .001, η^2^ = .61, *f =*1.24. The Progressive-generated gender critical faces (*M* = 3.02) were rated as *older* than the Traditional Liberal–generated faces (*M* = 3.94), *p <* .001. Conversely, for the self-identification stance there was a statistically significant effect in the opposite direction, where the Progressive-generated faces (*M* = 5.57) were rated as *younger* than the Traditional Liberal–generated faces (*M* = 4.99), *p <* .001. Finally, comparing the Progressive-generated concrete and abstract gender critical faces, we found that the face generated from the concrete exemplar (*M =* 3.17) was rated less *old* than the face generated from the abstract exemplar (*M =* 2.88), *f =*.19, *p <* .001.

##### Belief in Self-Identification

There was a significant main effect of gender identity stance, *F*(1, 299) = 209.17, *p* < .001, η^2^ = .41, *f =*.83. Overall, gender critical faces (*M =* 3.48) were judged as lower on *belief in self-identification* than those in favor of self-identification (*M =* 4.43). The two-way interaction between political identity of generator and gender identity stance was significant, *F*(1,299) = 75.07, *p*<.001, η^2^ = .20, *f =*.50. The Progressive-generated gender critical faces (*M* = 3.27) were rated as lower on *belief in self-identification* than the Traditional Liberal–generated faces (*M* = 3.68), *p <* .001. Conversely, for the self-identification stance there was a statistically significant effect in the opposite direction, where the Progressive-generated faces (*M* = 4.51) were rated as higher on *belief in self-identification* than the Traditional Liberal–generated faces (*M* = 4.34), *p <* .001. Finally, comparing the Progressive-generated concrete and abstract gender critical faces, we found that the face generated from the concrete exemplar (*M =* 3.44) was rated higher on *belief in self-identification* than the face generated from the abstract exemplar (*M =* 3.10), *f =*.24, *p <* .001.

#### Absolute Trait Ratings

As in Study 1, we also tested for absolute differences in traits (i.e., the extent to which the eight prototype faces would be seen as actively manifesting a given attribute). To achieve this, we computed a series of one-sample *t*-tests, Holm (1979) corrected, testing whether participants’ evaluations were significantly different from 4 (the scale midpoint). As seen in Table 4, regardless of condition (abstract or concrete), both Progressive and Traditional Liberal–generated self-identification faces were judged positively on the valenced traits (*warm, competent, moral*, *unprejudiced toward trans people*) and as *feminine*, *young* and *believing in self-identification*. Regarding the gender critical abstract faces, both the Progressive and Traditional Liberal–generated faces were judged negatively on the valenced traits (*cold, incompetent, immoral*, *prejudiced toward trans people*) as well as *old, right-wing* and *gender critical*. The Progressive-generated abstract gender critical face was also actively seen as *masculine*. For the concrete gender critical (J.K. Rowling) face, the Progressive-generated face was negatively evaluated (*cold, prejudiced toward trans people*), while the Traditional Liberal–generated face was evaluated positively on the valenced traits (*warm, competent, moral, unprejudiced toward trans people*). In case of the non-valenced attributes, the Progressive-generated J.K. Rowling face was rated as *old, right-wing, gender critical* but still *feminine*, while the Traditional Liberal–generated face was rated as *feminine, left-wing* and *young looking.*

#### Discussion

As expected, gender critical faces were evaluated more negatively than self-identification faces, consistent with left-wingers’ preference for a self-identification stance (H2). This was moderated by generator type; relative to those generated by Traditional Liberals, gender critical faces generated by Progressives were viewed more negatively (H3); conversely, among self-identification faces, the Progressive-generated faces were rated more positively. There was no support for H4. Contrary to expectations, the Progressive-generated concrete gender critical face (J.K. Rowling) was rated more favorably than the abstract gender critical face. This suggests that Progressives’ negative biases may manifest most strongly when projected onto an abstract description, rather than mitigated by pre-existing knowledge about a public figure’s appearance. Nevertheless, the Progressive-generated mental representation of J.K. Rowling was seen as actively cold, unlike the Traditional Liberal–generated Rowling face, which was seen as warm and competent.

The effect sizes for warmth and competence seem to indicate greater influence of warmth on evaluations, which may be due to the social nature of (in)group derogation (Hack et al., 2013) and the face-valid qualities of the gender critical face (e.g., frowning and unattractive). Nevertheless, effect sizes of pairwise comparisons indicate that we were able to replicate the main findings from Study 1: Progressives (vs. Traditional Liberals) generated a relatively negative face (in terms of warmth and competence) when presented with a concrete example of a public figure (J.K. Rowling) expressing gender critical views.

Most strikingly, we replicated our unexpected finding from Study 1, whereby by Traditional Liberals generated an actively positive depiction of J.K. Rowling. This is in stark contrast to the actively negative depiction of Rowling generated by Progressives, and the actively negative depiction that Traditional Liberals had generated of an unspecified other who expressed the same, in-group dissenting gender critical beliefs. These findings reinforce the notion that while Traditional Liberals may also derogate an in-group dissenter—albeit to a lesser extent than Progressives—there are qualities specific to J.K. Rowling that entirely mitigate these biases.

## General Discussion

Over the past decade, ideological symmetry approaches have called into question the long-standing assertion that out-group derogation is primarily a right-wing inclination, relative to ideological left-wingers typically characterized in terms of tolerance for divergent views (Crawford & Brandt, 2020). While left-wingers can be shown to derogate ideologically divergent others and views (Frimer et al., 2017), Proulx et al. (2023) demonstrated an asymmetry existing *within* self-identified left-wingers, whereby Progressives demonstrated more intolerance for an out-group (conservatives) relative to Traditional Liberals. In this paper, we tested whether this asymmetry in derogation would extend to a dissenting in-group member—specifically, a fellow left-winger expressing gender critical (vs. self-identification) views. Beyond the “culture wars,” relative tolerance for those with dissenting views on this issue has increasing impact on electoral coalitions across the Western left-wing (Lilla, 2018). The importance of understanding these divisions will only increase as public opinion rapidly evolves across the political spectrum (Smith, 2025).

Using J.K. Rowling as someone from the Left expressing gender critical (i.e., dissenting) views, Study 1 revealed that the classification image of Rowling generated by Progressives (compared to that of Traditional Liberals) elicited more negative evaluations from naïve raters. Moreover, Traditional Liberals generated actively flattering representations of Rowling. Study 2 showed that, overall, representations of gender critical faces were judged more negatively than self-identification representations, regardless of generators’ left-wing stance. This is in line with our left-wingers’ modal self-identification stance. Moreover, Progressive-generated gender critical faces were evaluated more negatively than those generated by Traditional Liberals, indicating greater relative intolerance of these dissenting views. Finally, we replicated the finding whereby Traditional Liberals generated an actively positive depictions of Rowling, despite being aligned with Progressives in generating a negative depiction of an abstract left-wing individual holding gender critical beliefs.

Indeed, across both studies (UK and US samples), Progressives (actively negative) and Traditional Liberals (actively positive) held diametrically opposing mental representations of J.K. Rowling. Importantly, these representations appear remarkably consistent, given the divergent demographic make-up of the UK (*M_age_* = 20.07 university students who were predominantly white women) and US (*M_age_* = 39.70 with greater diversity in education levels, race and gender) samples. This consistency speaks to the robustness of perceptions of Rowling across these left-wing identity kinds, suggesting that the divergent representations maintained by Progressives and Traditional Liberals may be differentially impacted by general negative discourse within the Western left-wing media (Ravell, 2023). While both left-wing identify kinds generated a negative image of an abstractly described gender critical left-winger, the relative proclivity of Traditional Liberals to take more factors into account when making social judgments (Hawkins et al., 2018) may have allowed for an actively positive appraisal of Rowling, compared to their Progressive counterparts.

Finally, Proulx et al. (2023) found that while both left-wing identity kinds generated negative representations of “Conservative” faces, Progressives (relative to Traditional Liberals) generated faces that were judged as even more unintelligent and incompetent, suggesting meaningful differences in the degree of derogation directed toward an out-group target. To our knowledge, the present studies are the first to demonstrate a valenced divergence of *kind* within the Left, which is made for a target *in-group* member, such that Progressives and Traditional Liberals generated actively negative versus positive representations of Rowling. To date, cultural commentators have anecdotally noted the divergence of opinion on Rowling within the Left, suggesting that the predominantly Progressive disdain for Rowling underlies failed efforts to broadly boycott products derived from her writing (e.g., Lewis, 2023). Our findings offer stark and compelling evidence for the reality of these divergent perceptions.

### Limitations and Future Directions

Relative tolerance and approbation for divergent gender identity views is one of the most publicly fractious issues across the cultural and political left-wing. It was for this reason that the present research focuses on this issue, one where Progressives show less tolerance of in-group dissent relative to Traditional Liberals. While the current findings are compelling, we cannot conclude that Progressives are *always* less tolerant of in-group dissenters relative to Traditional Liberals. Indeed, while both left-wing kinds derogated gender critical view-holders, it may be that exploring different issues would also allow us to specify whether the divergent impressions of Rowling are more a matter of heightened Traditional Liberal tolerance, or heightened Progressive moral outrage on the specific issue of gender identity. Further research may also examine generational, gender and race-based differences within the broader divide between Progressives and Traditional Liberals. While the findings across our demographically diverse samples were consistent, it may also be the case that samples allowing for robust analyses of representations specific to particular demographic characteristics (e.g., participant gender and age) might present distinct perceptions of Rowling maintained by Progressives on this particular social issue (Lewis, 2020).

With J.K. Rowling as our example of left-wing dissent, we chose a public figure with a long history of liberal advocacy, who was also explicitly noted as a fellow left-winger in Study 2. Nevertheless, it may be the case that Progressives’ heightened derogation of Rowling was associated with a relative *othering* of this target relative to Traditional Liberal appraisals, such that some Progressives may have judged Rowling as an out-group member on the issue of gender identity, rather than an in-group member based on her broader left-wing affiliation. While the findings nevertheless demonstrate an asymmetrical view of Rowling within these left-wing kinds, future research could examine the extent to which individual Progressives specifically contest the view that Rowling is an in-group member, and how such perceptions may impact mental representations of her.

In a similar vein, additional control or contrast conditions would allow us to further specify our effects. In both studies, we compared target appraisals in absolute terms against the scale midpoint, and in Study 2, we compared appraisals of gender critical faces against faces generated for self-identification targets. Nevertheless, future studies could compare both targets against a conceptually neutral baseline in terms of gender identity attitudes, for example, a target expressing ambivalence on the issue (Han et al., 2023), a relatively common view among people contemplating this complex social issue (Tryl et al., 2022). The inclusion of extra conditions could also compare or contrast our findings with “back sheep” effects (Marques et al., 1988), where an in-group dissenter (e.g., gender critical left-winger) is judged *more harshly* than an out-group member (e.g., conservative) expressing the same view. If the representation of a fellow left-winger holding divergent views was rated *more* negatively than a conservative, this would provide even stronger evidence of in-group derogation.

Taken together, considerations of participant demographics, “othering” as a derogation strategy and absolute (vs. relative) comparisons may offer an overall line of sight on another unanticipated finding: the progressive “masculinization” of Rowling we observe across studies.

In Study 1, we found that Progressives (vs. Traditional Liberals) generated a relatively less feminine depiction of Rowling, and in Study 2, an actively masculine depiction. It may be the case that the representative preponderance of women when sampling Progressives means that they may be more likely to “other” Rowling by, perhaps counterintuitively, diminishing their shared femininity after expressing gender critical views. Future research may explore this evocative possibility.

Finally, further research could also explore the extent to which Progressives and Traditional Liberals may differ in their propensity to reassess in-group dissenters. Prior research suggests that group members are more negative toward reintegrated deviant in-group members than those who remained excluded (Chan et al., 2010). On the issue of gender identity, future research could investigate how Progressives and Traditional Liberals react to scenarios where public left-wing dissenters have apologized or recanted their espousing of gender critical views (e.g., Irish singer Róisín Murphy; Beaumont-Thomas, 2023).

### Conclusion

In sum, this research has demonstrated asymmetry within the left in derogation toward a dissenting in-group member. Progressives hold relatively negative perceptions of left-wing dissenters on the issue of gender identity, regardless of whether the gender stance exemplar is presented in the abstract or as an actual person. However, the negative perception of J.K. Rowling appears unique to Progressives. An actively positive portrayal is held by Traditional Liberals, who appear to maintain a more “*benevolent, liberal exterior*” for Rowling, despite her gender critical views.

## Supplemental Material

sj-docx-1-psp-10.1177_01461672251378189 – Supplemental material for The Face of Left-Wing Dissent: Progressives and Traditional Liberals Generate Divergently Negative and Positive Representations of J.K. Rowling Supplemental material, sj-docx-1-psp-10.1177_01461672251378189 for The Face of Left-Wing Dissent: Progressives and Traditional Liberals Generate Divergently Negative and Positive Representations of J.K. Rowling by Elena A. Magazin, Geoffrey Haddock and Travis Proulx in Personality and Social Psychology Bulletin

sj-docx-2-psp-10.1177_01461672251378189 – Supplemental material for The Face of Left-Wing Dissent: Progressives and Traditional Liberals Generate Divergently Negative and Positive Representations of J.K. Rowling Supplemental material, sj-docx-2-psp-10.1177_01461672251378189 for The Face of Left-Wing Dissent: Progressives and Traditional Liberals Generate Divergently Negative and Positive Representations of J.K. Rowling by Elena A. Magazin, Geoffrey Haddock and Travis Proulx in Personality and Social Psychology Bulletin
