# Supplementary material for: The Face of Left-Wing Dissent: Progressives and Traditional Liberals Generate Divergently Negative and Positive Representations of J.K. Rowling
Source: Pers Soc Psychol Bull. 2025 Nov 5;52(11):3615–31. doi: 10.1177/01461672251378189 (PMC13646391; doi:10.1177/01461672251378189)
Supplement: Supplementary file 1 [file sj-docx-1-psp-10.1177_01461672251378189.docx]

**Appendix A. Instructions for Study 1**

**Appendix B.** Instructions Study 2 (Abstract)

**Appendix C.** Instructions Study 2 (Concrete – Lady Gaga)

**Appendix D.** Instructions Study 2 (Concrete – J.K. Rowling)
