# Supplementary material for: The Face of Left-Wing Dissent: Progressives and Traditional Liberals Generate Divergently Negative and Positive Representations of J.K. Rowling
Source: Pers Soc Psychol Bull. 2025 Nov 5;52(11):3615–31. doi: 10.1177/01461672251378189 (PMC13646391; doi:10.1177/01461672251378189)
Supplement: Supplementary file 2 [file sj-docx-2-psp-10.1177_01461672251378189.docx]

Supplementary Materials

Contents

[S1 FA Results and Zero-Order Correlations between PVS and the Rating Dimensions for Study 1	2](#_Toc207279464)

[S2 Estimation Statistics for Study 1	4](#_Toc207279465)

[S3 Exploratory Analysis for Attitude Measures in Study 2	6](#_Toc207279466)

[S4 FA Results Zero-Order Correlations between PVS and the Rating Dimensions for Study 2	8](#_Toc207279467)

[S5 Estimation Statistics for Study 2	10](#_Toc207279468)

[S6 Instructions for Study 1 and 2	15](#_Toc207279469)

# S1 FA Results and Zero-Order Correlations between PVS and the Rating Dimensions for Study 1

**Table S1.1**. *Factor Loadings for direct oblimin oblique rotated factor solution for Study 1*

| Rating item | Factor loadings | |
| --- | --- | --- |
|  | 1 | 2 |
| Factor 1: Warmth |  |  |
| Warm | **.900** |  |
| Likeable | **.861** |  |
| Attractive | **.545** |  |
| Factor 2: Competence |  |  |
| Competent |  | **.724** |
| Hardworking |  | **.713** |
| Intelligent |  | **.702** |

*Study 1*. From our total sample of 178, we selected those participants who had placed themselves as moderate or left-wing on the 7-point scale (1 *Left-wing*, 7 *Right-wing*; below or equal to four) and who selected either Traditional Liberal or Progressive on the Political Categorisation item. 110 participants remained and we calculated the PVS score (*M*=4.40, *SD*=.59). As seen in Table S1.2, there were no significant correlations between PVS and valance/non-valanced trait ratings of the Progressive or Traditional Liberal generated J.K. Rowling face.

**Table S1.2**. Zero-Order Correlations between PVS and the Rating Dimensions for each of the two J.K. Rowling prototype faces (*Study 1*)

| Trait |  |  |
| --- | --- | --- |
|  |  | J.K. Rowling |
|  | Generator | PVS |
| Warmth | TL | .08  [-.10,.27] |
|  | P | -.11  [-.29,.08] |
| Competence | TL | .12  [-.06,.31] |
|  | P | .03  [-.16,.21] |
| Moral | TL | . 07  [-.12,.25] |
|  | P | -.15  [-.33,.04] |
| Unprejudiced | TL | .11  [-.08,.29] |
|  | P | -.21  [-.38, -.02] |
| Feminine | TL | .15  [-.04,.33] |
|  | P | .20  [.02,.38] |
| Left-wing | TL | -.10  [-.28,.03] |
|  | P | .02  [-.17,.21] |
| Young | TL | .08  [-.11,.26] |
|  | P | -.10  [-.28,.09] |

Note: *TL=generated by Traditional Liberals, P= generated by Progressives*

# S2 Estimation Statistics for Study 1

In Figure S2, we can observe the effect sizes as bootstrapped 95% confidence intervals for paired sample t-tests (a) as well as one-sample t-tests (b). [Estimation statistics](https://en.wikipedia.org/wiki/Estimation_statistics) provides a unique data analysis method which aims to avoid some of the challenges of significance testing (Ho et al., 2019). It employs familiar statistical concepts (i.e., means, mean differences, and error bars) to generate results which focus on the effect size of the experiment, making them more robust. As such, we used bootstrap-coupled estimation via <https://www.estimationstats.com/#/>. As seen in Figure S2a, in line with our hypothesis, we found significant negative moderate-to-strong effects, whereby the Progressive-generated J.K Rowling face was rated *as colder and less competent* as well as being rated less *left-wing, feminine, moral, young and more prejudiced towards trans people* than the Traditional Liberal-generated face (*p<*.001). Furthermore, when compared to the scale mid-point (4), estimation statistics revealed opposite effects on evaluations for Progressives and Traditional Liberal generated J.K. Rowling face. The Progressive generated J.K Rowling face was seen as *cold, unattractive, feminine, right-wing, immoral, old* and *prejudiced against trans people* (*p*<.001). In contrast, the Traditional Liberal generated J.K. Rowling faces was seen as *warm*, *competent, feminine, moral, left-wing, young* and *unprejudiced towards trans people (p*<.001*)*.

**Figure S2**. *Effect sizes for paired-sample and one-sample bootstrapped coupled estimation for the Warmth, Competence and the 5 other traits per generator type, gender identity stance for the J.K Rowling face generation*

Note: S2a) *The paired Cohen's d for 7 comparisons are shown in the above Cumming estimation plot. Each paired mean difference is plotted as a bootstrap sampling distribution; S2b) The Cohen's d for 14 comparisons against the shared control M(4) are shown in the above Cumming estimation plot. Mean differences are depicted as dots and the curve indicates the resampled distribution of the mean difference; 95% confidence intervals are indicated by the ends of the vertical error bars. 5000 bootstrap samples were taken; the confidence interval is bias-corrected and accelerated. TL= generated by Traditional Liberals; P= generated by Progressives; From left to right, the different judgements (W=warmth, C=competence; F= feminine, LW= left-wing, Mo=moral, Y=young, Up=unprejudiced towards trans people).*

# S3 Exploratory Analysis for Attitude Measures in Study 2

To better understand Progressives’ and Traditional Liberals’ prior perceptions of those who are in favour of self-identification or gender critical (*abstract* or *concrete*) we pre-registered two exploratory attitude measures. A series of independent sample t-tests revealed that Progressives, relative to Traditional Liberals, had significantly more negative feelings towards someone described in abstract terms who is gender critical (*M_P_*=2.59, SD=1.44; *M_TL_*=4.02, SD=1.53; *t*(84)=4.38, *p*<.001, d=.95) and also felt they have a negative influence on society (*M_P_*=2.43, SD=1.64; *M_TL_*=3.88, SD=1.48; *t*(84)=4.41, *p*<.001, d=.96). We also found that Progressives had significantly more negative feelings towards J.K. Rowling than Traditional Liberals (*M_P_*=2.81, SD=1.47; *M_TL_*=3.84, SD=1.31; *t*(104)=3.82, *p*<.001, d=.75) and they also believed her to have more of a negative impact on society (*M_P_*=2.73, SD=1.39; *M_TL_*=3.84, SD=1.28; *t*(104)=4.28, *p*<.001, d=.84). Towards those holding a self-identification stance in both abstract and concrete terms (abstract: *M_P_*=6.00, SD=1.46; *M_TL_*=5.33, SD=1.78; *t*(89)=-1.98, *p*=.050, d=-.42; concrete: *M_P_*=5.88, SD=1.04; *M_TL_*=5.60, SD=1.05; *t*(91)=-1.26, *p*=.209, d=-.26), there was no significant difference in the attitudes held by Progressives and Traditional Liberals.. Finally, Progressives felt those holding an abstract self-identification stance had a more positive impact on society than Traditional Liberals did (*M_P_*=6.00, SD=1.40; *M_TL_*=5.28, SD=1.83; *t*(89)=-2.12, *p*=.036, d=-.45); but there was no difference in the impact they thought Lady Gaga had on society (concrete - *M_P_*=5.79, SD=1.15; *M_TL_*=5.69, SD=1.02; *t*(91)=-.46, *p*=.650, d=-.09).

*Absolute Judgement*

We tested for absolute differences in judgement (i.e., the extent to which Traditional Liberals and Progressives in each of the four conditions express positive or negative attitudes towards someone who is in favour of self-id or gender critical and described in either an abstract or concrete matter). To achieve this, we computed a series of one-sample *t*-tests, and tested whether participants’ evaluations were significantly different from 4 (the scale mid-point). As seen in Table S3, we found that both Progressives and Traditional Liberals actively hold positive feelings towards those are in favour of self-identification and Lady Gaga and believe such figures have a positive influence on society. However, while Progressives actively have negative feelings towards those who are gender critical and J.K. Rowling, Traditional Liberals seem to be neutral towards such figures (no significant difference from the midpoint).

**Table S3**. *Mean ratings, paired sample and one-sample t-test values for judgements of personal feelings towards and societal influence per generator type, gender identity stance and information type*

| Attitude | Trait |  | Comparison | | | Judgement | |
| --- | --- | --- | --- | --- | --- | --- | --- |
|  |  | Generator | *M* | *t* | Cohen’s *d* | One sample *t* (vs. 4) | Cohen’s *d* |
|  | SID Abstract | TL | 5.33 | -1.98 | -.42 | 4.88*** | .74 |
|  |  | P | 6.00 |  |  | 10.54*** | 1.37 |
| Positive feelings | SID Concrete (Lady Gaga) | TL | 5.60 | -1.26 | -.26 | 10.19*** | 1.52 |
|  |  | P | 5.88 |  |  | 12.56*** | 1.80 |
|  | GC Abstract | TL | 4.02 | 4.38*** | .95 | .09 | .01 |
|  |  | P | 2.59 |  |  | -5.10*** | -.97 |
|  | GC Concrete (JKR) | TL | 3.84 | 4.06*** | .75 | -.90 | -.12 |
|  |  | P | 2.81 |  |  | -5.97*** | -.81 |
|  | SID Abstract | TL | 5.38 | -2.112* | -.45 | 4.58*** | .70 |
|  |  | P | 6.00 |  |  | 10.70*** | 1.43 |
| Positive societal  influence | SID Concrete (Lady Gaga) | TL | 5.69 | -.46 | -.09 | 11.12*** | 1.66 |
|  |  | P | 5.79 |  |  | 11.38*** | 1.56 |
|  | GC Abstract | TL | 3.88 | 4.41*** | .96 | -.58 | -.08 |
|  |  | P | 2.43 |  |  | -5.51*** | -1.02 |
|  | GC Concrete (JKR) | TL | 3.84 | 4.29*** | .84 | -.92 | -.12 |
|  |  | P | 2.73 |  |  | -6.21*** | -.91 |

*Note: SID= in favour of self-identification, GC= gender critical; TL= Traditional Liberal; P=Progressive; ***p<.001*

# S4 FA Results Zero-Order Correlations between PVS and the Rating Dimensions for Study 2

**Table S4.1**. *Factor Loadings for direct oblimin oblique rotated factor solution for Study 2.*

| Rating item | Factor loadings | |
| --- | --- | --- |
|  | 1 | 2 |
| Factor 1: Warmth |  |  |
| Likeable | **.903** |  |
| Warm | **.886** |  |
| Attractive | **.799** |  |
| Factor 2: Competence |  |  |
| Competent |  | **.816** |
| Hardworking |  | **.670** |
| Intelligent |  | **.623** |

*Study 2.* From our total sample of 301, we selected those participants who had placed themselves as moderate or left-wing on the 7-point scale (1 *Left-wing*, 7 *Right-wing*; below or equal to four) and who selected either Traditional Liberal or Progressive on the Political Categorisation item. 204 participants remained and we calculated the PVS score (*M*=4.19, *SD*=.76). Table S4.2 shows the correlations between PVS and the individual evaluations for the attributes of the 8 protype faces. We found that, the higher one scores on the PVS, the more likely they were to judge the concrete gender critical (J.K. Rowling) face generated by Progressives as *less left-wing*. Meanwhile, the higher one is on the PVS, the more likely they were to rate the Traditional Liberal generated abstract gender critical face as *less left-wing*.

**Table S4.2**. Zero-Order Correlations between PVS and the Rating Dimensions for each of the 8 prototype faces (*Study 2*)

| Trait |  | Self-identification | | Gender Critical | |
| --- | --- | --- | --- | --- | --- |
|  |  | Abstract | Lady Gaga | Abstract | J.K. Rowling |
|  | Generator | PVS | PVS | PVS | PVS |
| Warmth | TL | .00  [-.13,.14] | -.06  [-.20,.08] | -.07  [-.20,.07] | -.11  [-.25,.02] |
|  | P | -.11  [-.24,.03] | -.11  [-.24,.03] | .02  [-.12,.16] | -.11  [-.24,.03] |
| Competence | TL | .05  [-.09,.18] | .08  [-.06,.22] | -.04  [-.18,.09] | -.01  [-.15,.12] |
|  | P | .05  [-.09,.18] | .04  [-.10,.17] | .01  [-.13,.14] | -.01  [-.15,.13] |
| Moral | TL | .07  [-.07,.20] | .10  [-.03,.24] | .09  [-.05,.22] | -.04  [-.18,.09] |
|  | P | .08  [-.06,.21] | .08  [-.05,.22] | .01  [-.13,.15] | -.08  [-.22,.06] |
| Unprejudiced | TL | .01  [-.12,.15] | -.01  [-.15,.13] | -.13  [-.26,.01] | -.07  [-.20,.07] |
|  | P | .04  [-.09,.18] | .05  [-.08,.19] | -.09  [-.23,.05] | -.12  [-.26,.01] |
| Belief in self-id | TL | .00  [-.13,.14] | -.02  [-.15,.12] | -.12  [-.25,.02] | -.09  [-.23,.04] |
|  | P | .05  [-.09,.19] | .02  [-.11,.16] | -.05  [-.19,.09] | -.13  [-.26,.01] |
| Feminine | TL | -.07  [-.21,.07] | -.01  [-.15,.12] | -.03  [-.17,.10] | -.03  [-.17,.11] |
|  | P | -.07  [-.21,.07] | -.05  [-.19,.09] | .01  [-.12,.15] | -.03  [-.17,.10] |
| Left-wing | TL | .06  [-.07,.20] | -.02  [-.16,.11] | **-.14***  [-.28, -.01] | -.10  [-.24,.03] |
|  | P | .13  [-.01,.26] | .01  [-.13,.14] | -.10  [-.23,.04] | **-.16***  [-.29, -.02] |
| Young | TL | .07  [-.07,.20] | .01  [-.12,.15] | -.10  [-.23,.04] | -.08  [-.22,.05] |
|  | P | .02  [-.12,.16] | .01  [-.13,.15] | -.05  [-.18,.09] | -.11  [-.25,.02] |

Note: *TL=generated by Traditional Liberals, P= generated by Progressives;* **p*<.05

# S5 Estimation Statistics for Study 2

Figure S5 shows the estimation plots of the effect sizes corresponding to the analysis the simple pairwise comparisons of the interaction between gender identity stance and generator type for each judgement, generated using bootstrapped-coupled estimation as for Study 1.

*Warmth*. There was a large, statistically significant effect which shows that Progressive generated gender critical faces were rated as *colder* than the Traditional Liberal-generated faces, *p<*.001. This supports H3. Conversely, for the self-identification stance there was a statistically significant moderate effect in the opposite direction, where the Progressive-generated faces were rated as *warmer* than the Traditional Liberal-generated faces, *p<*.001.

*Competence*. There was a moderate, statistically significant effect which shows that Progressive generated gender critical faces were rated as less *competent* than the Traditional Liberal-generated faces, *p<*.001. This supports H3. Conversely, for the self-identification stance there was a statistically significant small-to-moderate effect in the opposite direction, where the Progressive-generated faces were rated as more *competent* than the Traditional Liberal-generated faces *p<*.001.

*Moral*. There is a small-to-moderate, statistically significant effect which shows that Progressive generated gender critical faces were rated as less *moral* than the Traditional Liberal-generated faces, *p<*.001. This supports H3. Conversely, for the self-identification stance there was a statistically significant small-to-moderate effect in the opposite direction, where the Progressive-generated faces were rated as more *moral* than the Traditional Liberal-generated faces, *p<*.001.

*Unprejudiced towards trans people*. There was a moderate, statistically significant effect which shows that Progressive generated gender critical faces were rated as less *unprejudiced towards trans people* than the Traditional Liberal-generated faces, *p<*.001. This supports H3. Conversely, for the self-identification stance there was a statistically significant small-to-moderate effect in the opposite direction, where the Progressive-generated faces, were rated as more *unprejudiced towards trans people* than the Traditional Liberal-generated faces, *p<*.001.

*Feminine*. There is a large, statistically significant effect which shows that Progressive generated gender critical faces were rated as less *feminine* than the Traditional Liberal-generated faces, *p<*.001. This supports H3. Conversely, for the self-identification stance there was a statistically significant moderate effect in the opposite direction, where the Progressive-generated faces were rated as more *feminine* than the Traditional Liberal-generated faces, *p<*.001.

*Left-wing*. There was a moderate, statistically significant effect which shows that Progressive generated gender critical faces were rated as less *left-wing* than the Traditional Liberal-generated faces, *p<*.001. This supports H3. Conversely, for the self-identification stance there was a statistically significant moderate effect in the opposite direction, where the Progressive-generated faces were rated as more *left-wing* than the Traditional Liberal-generated faces, *p<*.001.

*Young*. There was a strong, statistically significant effect which shows that Progressive generated gender critical faces were rated as *older* than the Traditional Liberal-generated faces, *p<*.001. This supports H3. Conversely, for the self-identification stance there was a statistically significant moderate effect in the opposite direction, where the Progressive-generated faces were rated as *younger* than the Traditional Liberal-generated faces, *p<*.001.

*Belief in self-identification*. There was a moderate, statistically significant effect which shows that Progressive generated gender critical faces were rated as lower on *belief in self-identification* than the Traditional Liberal-generated faces, *p<*.001. This supports H3. Conversely, for the self-identification stance there was a statistically significant small effect in the opposite direction, where the Progressive-generated faces were rated as higher on *belief in self-identification* than the Traditional Liberal-generated faces, *p<*.001.

**Figure S5**. *Effect sizes and 95% CI and p-values for the simple pairwise comparisons of the interaction between gender identity stance and generator type for each judgement*

Note: *The paired Cohen's d for 16 comparisons are shown in the above Cumming estimation plot. Each paired mean difference is plotted as a bootstrap sampling distribution. Mean differences are depicted as dots and the curve indicates the resampled distribution of the mean difference; 95% confidence intervals are indicated by the ends of the vertical error bars. 5000 bootstrap samples were taken; the confidence interval is bias-corrected and accelerated. SeTL= self-identification face generated by Traditional Liberals; SeP= self-identification face generated by Progressives; GCTL= gender critical face generated by Traditional Liberals; GCP= gender critical face generated by Progressives; From left to right, the different judgements (W=warmth, C=competence; F= feminine, LW= left-wing, Mor=moral, Y=young, Up=unprejudiced towards trans people, SID=belif in self-identification).*

**Table S5.1**. *Cohen’s d, 95% CI and p-values for the simple pairwise comparisons of the interactions by generator type*

Note: *The paired Cohen's d are along with the 95% confidence intervals are indicated. 5000 bootstrap samples were taken; the confidence interval is bias-corrected and accelerated. TL=generated by Traditional Liberals; P= generated by Progressives*

| Trait | Self-identification | | | | | Gender Critical | | | |
| --- | --- | --- | --- | --- | --- | --- | --- | --- | --- |
|  |  | Abstract | | Lady Gaga | | Abstract | | J.K. Rowling | |
|  | Generator  comparison | *d[95%CI]* | *p* | *d [95%CI]* | *p* | *d [95%CI]* | *p* | *d [95%CI]* | *p* |
| Warmth | P-TL | .92[0.77,1.06] | <.001 | -.02[-.11, .06] | .591 | -.17[-.28, -.06] | .001 | -1.42[-1.59, -1.24] | <.001 |
| Competence | P-TL | .52[.39,.64] | <.001 | .02[-.07,.10] | .674 | -.20 [-.31, -.07] | <.001 | -.66 [-.80, -.54] | <.001 |
| Moral | P-TL | .26[.12,.39] | <.001 | .13[.04,.23] | .010 | -.14[-.26,.00] | .040 | -.44[-.58, -.30] | <.001 |
| Unprejudiced | P-TL | .41[.28,.55] | <.001 | -.00[-.11,.09] | .900 | -.13[-.25, -.01] | .023 | -.68[-.82, -.53] | <.001 |
| Feminine | P-TL | .71[.57,.85] | <.001 | .04[-.04,.11] | .298 | -.87[-1.03, -.71] | <.001 | -.98[-1.14, -.83] | <.001 |
| Left-Wing | P-TL | .34[.19,.49] | <.001 | .10 [-.00,.19] | .059 | -.15[-.28, -.02] | .023 | -.55[-.70, -.38] | <.001 |
| Young | P-TL | 1.09[.931.25] | <.001 | -.07[-.16,.02] | .109 | -.12[-.24,.00] | .052 | -1.50[-1.69, -1.30] | <.001 |
| Belief in self-id | P-TL | .37[.23,.50] | <.001 | -.05[-.16, .05] | .360 | -.09[-.21, .02] | .112 | -.64[-.78, -.49] | <.001 |

**Table S5.2**. *Cohen’s d, 95% CI and p-values for the simple pairwise comparisons of the interactions by information type*

Note: *The paired Cohen's d are along with the 95% confidence intervals are indicated. 5000 bootstrap samples were taken; the confidence interval is bias-corrected and accelerated. TL=generated by Traditional Liberals; P= generated by Progressives. Conc=concrete exemplar; Abs=abstract exemplar.*

| Trait | Self-identification | | | | | Gender Critical | | | |
| --- | --- | --- | --- | --- | --- | --- | --- | --- | --- |
|  |  | TL | | P | | TL | | P | |
|  | Information  type | *d[95%CI]* | *p* | *d [95%CI]* | *p* | *d [95%CI]* | *p* | *d [95%CI]* | *p* |
| Warmth | Conc-Abs | .76[0.61,.89] | <.001 | -.21[-.32, -.10] | <.001 | 1.76[1.55, 1.95] | <.001 | .52 [.38, .65] | <.001 |
| Competence | Conc-Abs | .39 [.26,.52] | <.001 | -.11[-.21,.01] | .037 | .88 [.73, 1.03] | <.001 | .43 [.28, .56] | <.001 |
| Moral | Conc-Abs | -.01[-.14,.12] | .834 | -.13 [-.24, -.02] | .003 | .58[.42, .74] | <.001 | .31[.17, .46] | <.001 |
| Unprejudiced | Conc-Abs | .22[.07,.36] | .003 | -.21[-.33, -.11] | .900 | .84[.68, .99] | <.001 | .33[.18, .46] | <.001 |
| Feminine | Conc-Abs | .83[.68, .96] | <.001 | .14[.06,.23] | .001 | 1.24[1.07, 1.40] | <.001 | 1.15[.97, 1.32] | <.001 |
| Left-Wing | Conc-Abs | .17[.01, .32] | .023 | -.08 [-.21, .03] | .105 | .60[.42, .75] | <.001 | .23[.09, .37] | <.001 |
| Young | Conc-Abs | .92[.78, 1.06] | <.001 | -.25 [-.35, -.14] | .109 | 1.68[1.49, 1.89] | <.001 | .25[.10, .39] | <.001 |
| Belief in self-id | Conc-Abs | .24[.10, .38] | .001 | -.17[-.30, -.06] | .003 | .80[.65, .96] | .112 | .29[.15, .42] | <.001 |

# S6 Instructions for Study 1 and 2

**S6A. Instructions for Study 1**

**S6B.** Instructions Study 2 (Abstract)

**S6C.** Instructions Study 2 (Concrete – Lady Gaga)

**S6D.** Instructions Study 2 (Concrete – J.K. Rowling)
